# Exceptionally long-range haplotypes in *Plasmodium falciparum* chromosome 6 maintained in an endemic African population

**DOI:** 10.1186/s12936-016-1560-7

**Published:** 2016-10-21

**Authors:** Alfred Amambua-Ngwa, Bakary Danso, Archibald Worwui, Sukai Ceesay, Nwakanma Davies, David Jeffries, Umberto D’Alessandro, David Conway

**Affiliations:** 1 Medical Research Council, Gambia Unit, Atlantic Road, Fajara, P.O. Box 273, Banjul, The Gambia; 2 London School of Hygiene and Tropical Medicine, Keppel Street, London, UK

**Keywords:** Malaria, Microsatellites, Selective sweep, Haplotypes, Drug resistance

## Abstract

**Background:**

Previous genome-wide analyses of single nucleotide variation in *Plasmodium falciparum* identified evidence of an extended haplotype region on chromosome 6 in West Africa, suggesting recent positive selection. Such a pattern is not seen in samples from East Africa or South East Asia, so it could be marking a selective process specific to West Africa. Analyses of the haplotype structure in samples taken at different times could give clues to possible causes of selection.

**Methods:**

This study investigates chromosome 6 extended haplotypes in The Gambia by analysing alleles at multiple microsatellite loci using genome sequence data previously obtained from clinical isolates collected in 2008, followed by genotyping of 13 loci in 439 isolates from 1984, 1991, 2008 and 2014. Temporal changes in haplotype structure and frequencies were determined.

**Results:**

A region of high linkage disequilibrium spanning over 170 kilobases (kb) was identified with both NGS and laboratory determined microsatellite alleles. Multiple long haplotypes were found in all temporal populations from The Gambia. Two of the haplotypes were detected in samples from 1984 and 1991. The frequency of long-range haplotypes increased in 2008 and 2014 populations. There was higher Fst between older and more recent populations at loci in proximity to genes involved in drug metabolism pathways.

**Conclusions:**

The occurrence of several long haplotypes at intermediate frequencies suggests an unusual mode of selection in chromosome 6, possibly combined with recombination suppression on specific haplotypes. Such selection apparently occurred before the emergence of known anti-malarial drug resistance alleles, and could be due to effects of other drugs or unknown processes that have long been operating in this endemic region.

**Electronic supplementary material:**

The online version of this article (doi:10.1186/s12936-016-1560-7) contains supplementary material, which is available to authorized users.

## Background

Adaptation of malaria parasites to various challenges, including human immune responses, drugs and other interventions has left genetic signatures in the *Plasmodium falciparum* genome [1]. These can be detected with polymorphic genetic markers showing reduced variability, increased linkage disequilibrium, extended haplotypes, excess of rare alleles or high frequencies of derived alleles at loci close to the target of selection [2, 3]. Anti-malarial drugs in particular exert a strong positive selection on the genome and several of the strongest signatures of directional selection are linked to drug resistance genes [4, 5]. Apart from these, a number of other *P. falciparum* genomic loci show evidence of positive selection in endemic populations [6, 7]. Some signatures of selection are apparent only in specific endemic populations, including an extensive selective sweep on chromosome 6 that was first described by analysing single nucleotide polymorphisms (SNPs) in parasites from Senegal and The Gambia in West Africa [6]. The evidence of extended haplotype homozygosity in chromosome 6 appears strong in West African parasite populations but weak or absent elsewhere [6, 8–11]. The size of the chromosomal region affected may be up to ~300 kb, and none of the ~60 genes in this region is a known direct target of selection from anti-malarial drugs. This result was obtained with SNP markers, but analysis of microsatellites may offer additional information due to their higher levels of polymorphism [12–14]. Microsatellite approaches are also attractive here because they require technology that is more accessible to low and medium income country labs. This allows for local targeted analysis of sweeps following initial scans with high density SNPs, which requires computational capacity that is limited in most of sub-Saharan Africa.

This study focuses on characterising the structure of *P. falciparum* chromosome 6 extended haplotypes around the selective signature in The Gambian population. This population benefits from prior knowledge of selective signatures from SNP studies, availability of whole genome short read sequence data for mining other polymorphisms such as microsatellites, and archived samples from the period prior to widespread chloroquine resistance. Microsatellite polymorphisms within the chromosome 6 region were targeted for this temporal analysis thanks to their abundance and sensitivity in determining haplotype structure around selective sweeps [14, 15]. The analysis of sequence data enabled the design of new laboratory genotyping assays, which focused on a set of 13 microsatellite loci across the region of extended haplotype structure.

## Methods

### *Plasmodium falciparum* isolates

This study analysed four sets of *P. falciparum* isolates from the Gambia. The first two comprises 56 and 67 isolates collected in 1984 and 1991 respectively around Farafenni, located in the middle of the country [4, 26]. During this period, chloroquine was still efficacious against most infections and resistance alleles were rare. The third population includes 166 isolates from the Greater Banjul area, in the west of the country, collected in 2008 when malaria transmission had decreased substantially and the first line treatment for malaria had been changed from sulfadoxine–pyrimethamine (SP) to artemether–lumefantrine (AL) [27]. The fourth was a recent population sample of 150 isolates collected in 2014 transmission season from the Greater Banjul area in the West, as well as Basse in the east of the country. Of the isolates collected in 2008, 76 had whole genome short sequence reads for mining microsatellite polymorphisms [16]. Single nucleotide polymorphisms and signatures of selection in this population has been described in a number of previous studies [4, 7, 16].

### Microsatellite scoring across *P. falciparum* chromosome 6 using Illumina short-read sequence data

Microsatellite markers across chromosome 6 of *P. falciparum* were determined from Illumina generated whole genome short sequence read data of the 68 isolates collected in 2008 as described previously [28]. For microsatellite identification and genotyping, short sequence reads were mapped against the *P. falciparum* 3D7 reference chromosome 6 sequences (version3, October 2012 release) to generate sequence alignment files (Bam) as previously described. Bam files were then screened for microsatellites against a reference library indexing all possible microsatellite repeats in the Pf3D7 reference chromosome 6 sequence created with the tandem repeat finder software (trf) and SciRoKo for perfect repeats [29, 30]. Algorithms for calling microsatellite alleles are as described for the programs RepeatSeq and Genotan [31, 32]. Quality settings for calling microsatellite genotypes incorporated a short read quality score of Q30 and read coverage across the microsatellite of 5X spanning a unique region bordering both sides of the locus. Allele calls were corrected to lengths reflective of the repeat unit assuming a stepwise mutation model in Microsatellite Analyzer package [33]. Perfect repeat tracts with number of repeats from 14 to 63 were chosen for further analysis. The final dataset employed included loci that were polymorphic and with less than 30 % missing calls. The objective was to identify a set of polymorphic markers from Illumina short sequence reads and select a subset for further analysis by PCR fragment sizing following amplification of DNA from the target populations.

### Selected chromosome 6 microsatellite loci for laboratory genotyping

Microsatellite loci employed in retrospective analysis of chromosome 6 selective sweep were selected from polymorphic loci determined from short read sequence data analysed for chromosome 6 as described above. These loci were perfect microsatellites, having at least two alleles in the Gambian population and included di- to hexa-nucleotide repeats. These were physically spaced at an average distance of ~15 kb across a 179.5 kb region, between positions 1069 and 1249 kb of the chromosome. Primer pairs were designed automatically for flanking sequences of each unique microsatellite locus with Primer3 software implemented in BatchPrimer using the Pf3D7 reference sequence as a template (Additional file 1: Targeted_13_ssr_stats). Primers for amplification and fragment labelling with 6FAM, TET, HEX and TAMRA dyes were commercially synthesized (Metabion).

### PCR amplification and capillary electrophoresis

For each sample, DNA was obtained from whole blood aliquot using the QiAmp DNA extraction kit. Amplification of targeted microsatellite loci followed a two round PCR reaction in which the first round was a multiplex for up to 3 loci in a 10 µL reaction mix with 0.2 µM of each outer primer pair, 1 µL of DNA and 1X PCR multiplex mix (Qiagen). Each amplification batch included the reference *P. falciparum* 3D7 DNA as a positive control and nuclease free water as a negative control. Thermocycling was achieved with a touchdown PCR protocol on a Q-cycler (Quantarus); initial denaturation at 95 °C for 5 min, touchdown from 65 to 55 °C at 1 °C/cycle followed by 25 cycles of 95 °C for 30 s, 54 °C for 1 min, 72 °C for 30 s. Final elongation was at 68 °C for 30 min. First round PCR reactions were used immediately for second rounded amplification with labelled primers or stored at 4 °C until needed. Second round PCR reactions were run separately for each locus using 1 µL of first round PCR product, 150 nM of each primer and 1X of MyTaq amplification mix (Bioline) in a 10 µL reaction. Amplification conditions were initial denaturation at 95 °C for 30 s, 25 cycles of 95 °C for 30 s, 54 °C for 30 s, 68 °C for 30 s. Final elongation was at 68 °C for 20 min. For capillary electrophoresis, round 2 PCR products were constituted at 1:50 dilution in 10 µL assay mix of HIDI formamide with GeneScan™ 600LIZ^®^ size standard. This was denatured by heating at 95 °C for 3 min and immediately cooled on ice. Labelled microsatellite fragments were separated on 3130xL DNA analyser. DNA fragment analysis traces for loci were processed in GeneMapper 4.1 and allele sizes were called using GeneMarker 1.1. Allele sizes were checked for error and binned using Tandem2 software, which corrects for deviations in expected fragment size based on repeat unit length of the target microsatellite. Binned genotypes were employed in determining pairwise haplotype frequencies and Linkage disequilibrium between contiguous loci using PowerMarker Version 3.5 and Midas. Fragment sizes for SSR1, SSR9 and SSR15 were correlated with illumina determined repeat length genotypes (Additional file 2).

### Population genetic analysis of chromosome 6 microsatellite loci

The allele frequencies, allelic richness, variability, expected heterozygosity, and Weir and Cockerham’s Fst for binned microsatellite were calculated with the heirfstat R package. Virtual heterozygosity (HE) was used to measure the overall genetic diversity at each locus. It is defined as, n/(n − 1)(1 − Σpi^2^), where n is the number of isolates analysed and pi is the frequency of the i-th allele in the population. To test which microsatellite loci showed population variation that deviated from neutral expectations, simulations of the distribution of pairwise Fst coefficient were carried out in Bayescan 2.1. Bayescan uses posterior probabilities to control for False Discovery rates (FDR), which is the proportion of false positives among candidate outlier loci. Simulations were performed at default settings; 100,000 iterations, 5000 pilot runs and 50,000 burn-in length. P values (one-sided) were estimated from 10,000 permutations of population assuming a panmictic null hypothesis. The FDR expressed as q value (FDR analogue of p value defined under multiple testing) was plotted against Fst per selected loci using Bayescan plot code in R to detect and list outlier loci at a 5 % FDR. Unique multilocus genotypes were determined using R allele Match unique package with a maximum mismatch setting at 3 out of 13 loci following mismatch and multiple match minimization. Partially matched haplotypes were further analysed with Haplotype Analysis 4.05. Visualization of the evolution of frequency of the common long range haplotype was done with the REHH package in R. For this, the loci with the lowest and highest Fst values within the window of high linkage disequilibrium were chosen as focal sites for determining the extent of haplotypes around them. To allow for REHH analysis, the fragment sizes were converted into a binary format in which ‘2’ represented the dominant allele while ‘1’ was for any other fragment length at this locus. Analysis and plotting were as described in the REHH manual.

## Results

### Diversity of chromosome 6 microsatellite loci derived from short sequence reads of *P. falciparum*

**Fig. 1:**
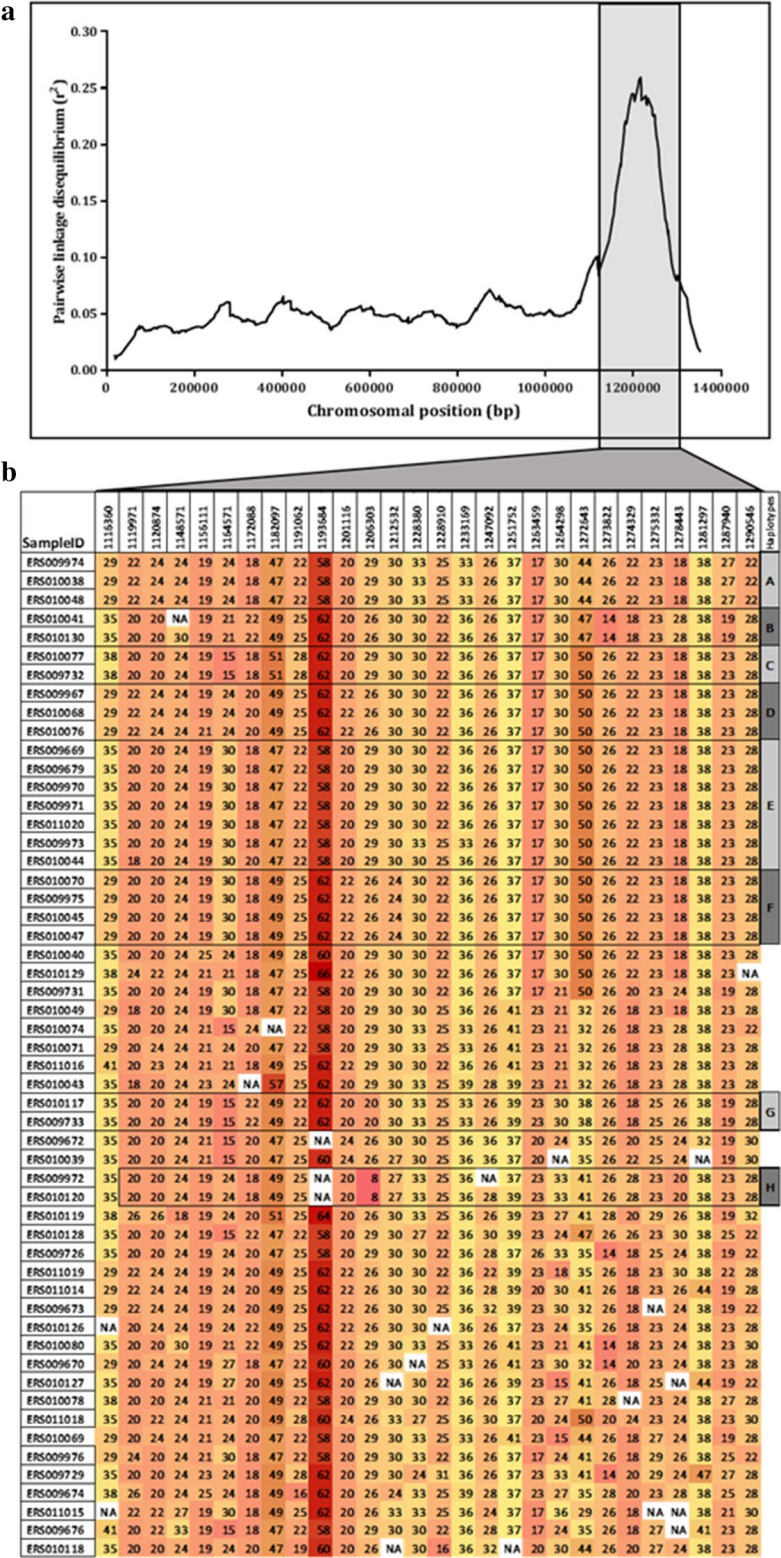
Microsatellite linkage disequilibrium and haplotypes across chromosome 6 of *Plasmodium falciparum* from the Gambia in 2008. **a** Mean pairwise linkage disequilibrium (r^2^) plotted against the physical position (base pairs on chromosome) of 775 polymorphic repeat loci across chromosome 6. Microsatellite genotypes were derived from short sequence reads of 54 *P. falciparum* isolates collected in 2008. The *shaded box* delineates the region of peak LD, representing the chromosome 6 selective sweep. **b** Zoom-in on 174 kb region of peak LD on panel ‘**a**’, showing the haplotypes from alleles (repeat-number) of 28 polymorphic microsatellite loci with at least three alleles. The *first column* in panel ‘**b**’ is the European Nucleotide Archive sample/data ID and the following columns are physical positions (bp) of chosen repeat loci on the chromosome. The *numbers* in cells are the repeat lengths and *colour* intensity for each cell in a column increases with increase in repeat lengths. Haplotypes defined by matching of loci alleles across the region are shown as grouped cells shaded with alternative intensities of *grey* and named arbitrarily from A to H

The number of repeats for 775 polymorphic microsatellite loci in chromosome 6 of *P. falciparum* were successfully scored from Illumina short read sequences of 54 out of 68 clinical isolates collected in 2008. The mean virtual heterozygosity for the 13 microsatellite loci was 0.47. Pairwise linkage disequilibrium, expressed as mean r^2^ values between all pairs of loci within a window of 10 kb containing at least 5 loci, ranged from 0 to 0.3. The values were significantly higher towards the 3′ end of the chromosome, mapping between positions 1100–1300 kb with a peak at ~1200 kb (Fig. 1a). Mean pairwise haplotype frequencies between loci in 10 kb windows across the chromosome showed a narrower peak of ~100 kb (1150–1250 kb) at this region on the chromosome (Additional file 3).

Long-range haplotypes spanning 184 kb were identified with microsatellite genotypes for 28 polymorphic loci across the region of peak LD for 54 isolates (Fig. 1b). These long-range haplotypes shared by at least 2–8 isolates were represented in 28 isolates (50 %). Following allele matching and haplotype extension from 12 loci within a window of 72.7 kb (1,201,161–1,273,822 bp) with the most intact haplotypes, 8 major long-range haplotypes at frequencies between 3.7 and 16.7 % were identified. The rest of the isolates had partial and recombinant forms of this haplotype.

### Analysis of microsatellite loci in temporal populations from The Gambia

**Fig. 2:**
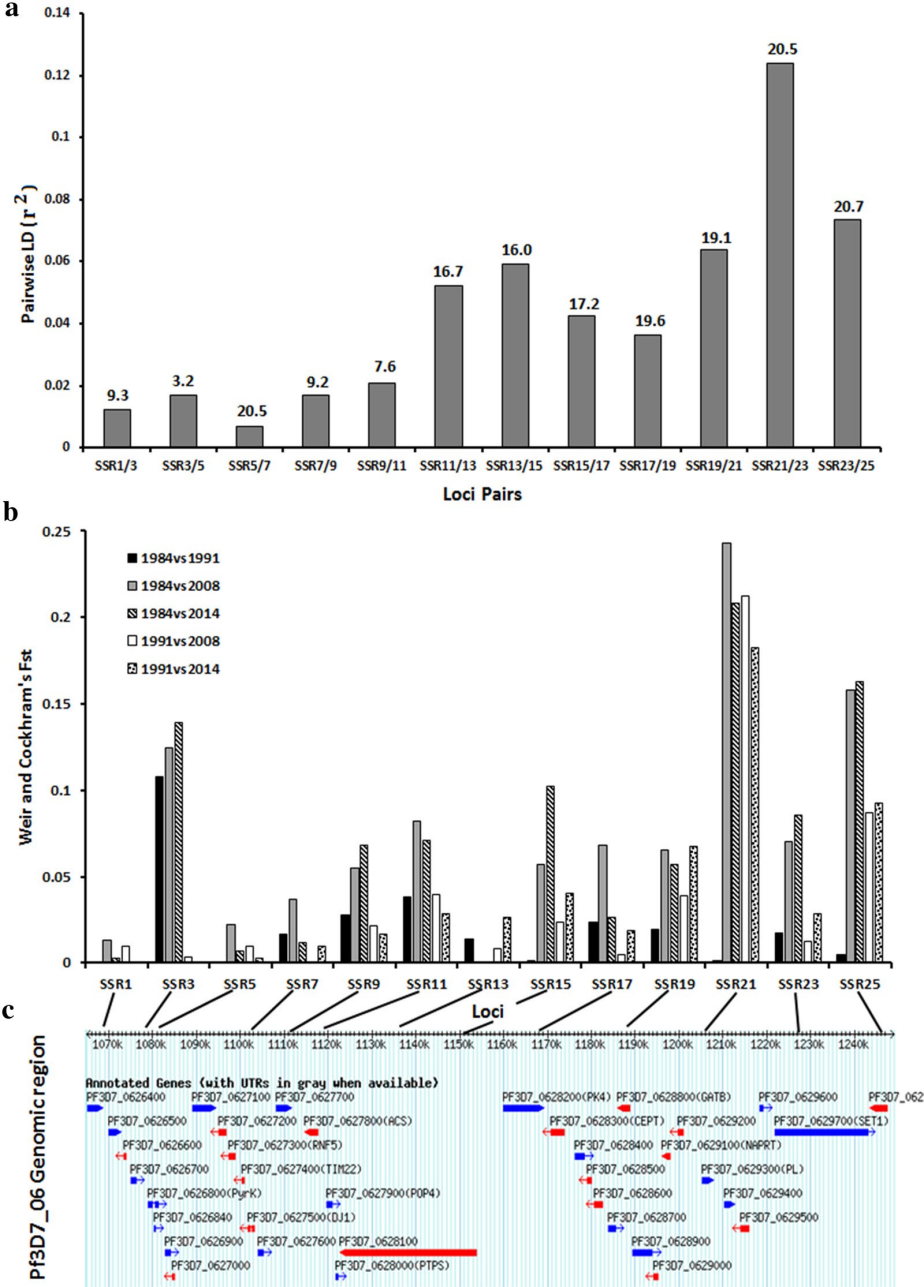
Analysis of genotypes at 13 microsatellite loci chosen across the 179.5 kb signature of selection on chromosome 6 of *P. falciparum*. **a** Extent of pairwise linkage disequilibrium (r^2^) between adjacent loci as columns labelled on the x-axis with the arbitrary names of the loci pair. The physical distance (kb) separating the pair of loci for which the r^2^ was determined is shown on *top* of each column. **b** Weir and Cockeram’s Fst at each locus for pairs of temporal populations compared. *Each column* represents the extent of Fst between population pairs. **c** *Snapshot* of genomeview display in PlasmoDB for the region covered by the 13 loci (http://www.plasmoDB.org). The connecting lines from locus names in panel ‘**b**’ x-axis maps the physical positions (kb) of each locus on the genome ruler in panel ‘**c**’. The *blue* and *red bars* in panel ‘**c**’ represent coding sequences with the *arrow head* showing the direction of translation. The gene ID of each coding sequence is shown above each *bar*

Laboratory genotyping was performed on 13 loci spanning 179.5 kb of the selective sweep region for 439 isolates from population samples taken in 1984 (N = 56), 1991 (N = 67), 2008 (N = 166) and 2014 (N = 150). These loci were separated by a mean distance of 15 kb (3.2–20.7 kb). The mean virtual heterozygosity across all loci was 0.77, ranging from 0.47 to 0.92 (highest in the dinucleotide repeat loci and decreasing with increasing repeat unit size). Pairwise linkage disequilibrium between adjacent loci revealed two peaks with r^2^ values of 0.06 and 0.12 respectively for loci SSR13 against SSR15 separated by 16 kb (1,135,849–1,151,868 bp) and for loci SSR21 against SSR23 separated by 20.5 kb (1,207,751–1,228,301 bp), (Fig. 2a). Per locus pairwise population Fst values were generally low (0–0.25). Higher indices were observed when comparing 1984 and 1991 to more recent populations from 2008 and 2014 for SSR3 (1,078,732 bp), SSR21 (1,207,751 bp) and SSR25 (1,249,025 bp), (Fig. 2b). Values between 1894 and 1991 were low for all loci except for SSR3. The lowest level of differentiation was between 2008 and 2014 populations, with nine out of 13 loci showing non-significant Fst values (Additional file 1: Tables-sheet: Fst-13Loci). Following Bayesian simulation of pairwise population Fst distribution at each locus, seven (SSR1, SSR3, SSR5, SSR7, SSR11, SSR13 and SSR19) out of the selected 13 loci had a pattern of variation between populations that deviated from neutral expectations (Additional file 4a). Of these, SSR13 (Pf3D7_06_v3: 1135849) had the strongest signature of deviation from neutrality. This locus is located within the Pf3D7_0628100 sequence, which codes for 6-pyruvoyltetrahydropterin synthase (PTPS). The other six loci had lower Fst and high q-values. Three loci: SSR19 (Pf3D7_06_v3:1188628), SSR21 (Pf3D7_06_v3:1207757) and SSR23 (Pf3D7_06_v3:1228301), also showed significant deviation of Fst and heterozygosity than expected under neutrality (Additional file 4b). These loci span the region of peak LD detected (Fig. 2a). There are 11 genes encoded in the region with highest LD and Fsts, which includes 3 transferases, a transportin, a transporter, a synthetase, and a synthase (Fig. 2c; Additional file 1: Tables-sheet: Genes_in_Sweep). The entire 179.5 kb region analysed with 13 microsatellite loci across temporal populations, codes for 36 genes, which are involve in a variety of metabolic processes (Additional file 1: Tables-sheet: Genes_in_Sweep).

### Shared long-range haplotypes

**Fig. 3:**
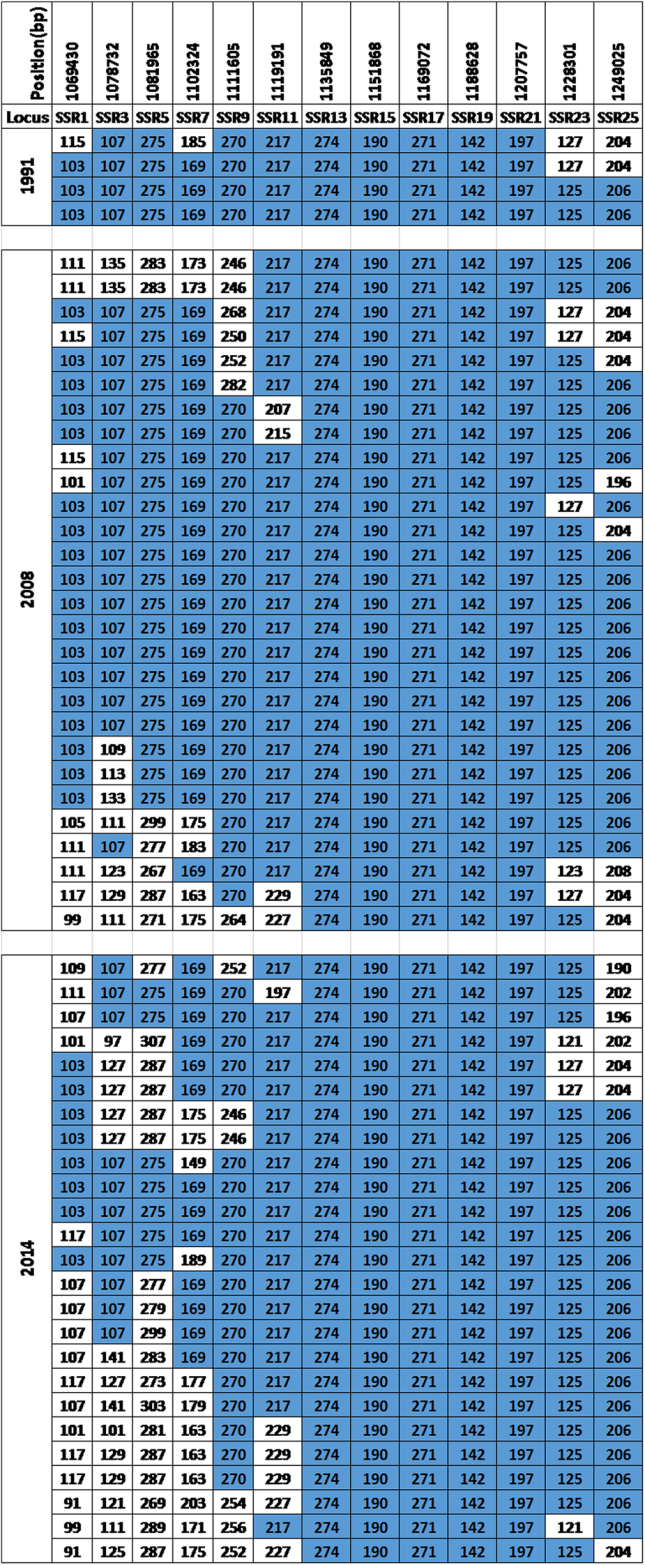
Structure of the dominant haplotype derived from fragment analysis of 13 microsatellite loci spanning 179.5 kb of chromosome 6 selective signature in *P. falciparum*. The *first row* shows the physical positions (bp) of each locus and the arbitrary names of the loci are presented in each column of the *second row*. The years of sampling for each population is shown in *merged rows* of the first column while the following columns are microsatellite loci positions on the chromosome. For each population, the rows present the haplotype of a sample across the 13 loci. The *numbers* in each cell is the allele (fragment size in bp determined from capillary electrophoresis) for a sample at each locus. Alleles of the major haplotype are shaded in *blue*

With complete matching at all 13 loci, 243 (55.4 %) isolates had unique haplotypes while 196 were shared by at least 2 isolates from the four temporal populations. Of the latter, 18 isolates shared the longest uninterrupted haplotype only from the 1991, 2008 and 2014 populations (Fig. 3). Recombinant and partial forms of this major haplotype were found in 39 other isolates from the same populations. The structure of this long-range haplotype was most conserved at five contiguous loci (SSR13-SSR21) spanning 71.9 kb (1,135,849–1,207,757 bp), (Fig. 3). With complete matching at all five loci within this conserved window, 4 (5.9 %), 28 (16.9 %) and 25 (16.6 %) isolates from 1991, 2008 and 2014 respectively had this haplotype group. This represents 13 % of all isolates from these populations (Fig. 3). There were 40 other haplotypes shared by 2–13 isolates from the four populations. Three of these haplotypes were present in the 1984 population of which 2 were seen in single isolates from 1991 and 2008 (Table 1). Nine haplotypes from 1991 were present at higher frequencies in 2008 and 2014.

**Table 1:** Frequencies of core haplotypes spanning 71.5 kb across the selective sweep region on chromosome 6 of *P. falciparum* from the Gambia collected in 1984, 1991, 2008 and 2014

Haplotype	1984	1991	2008	2014	Frequency
274 190 271 142 197		4	28	25	13.0
280 181 277 144 197			7	6	3.0
274 190 271 144 197		1	7	1	2.1
274 190 275 144 197				8	1.8
280 181 279 142 197			6	1	1.6
274 181 269 142 197		1	5		1.4
274 181 271 142 197		1	3	2	1.4
274 181 281 138 225			3	2	1.1
274 184 285 126 197		1	2	2	1.1
274 181 269 140 197			4		0.9
274 181 285 138 197		1		3	0.9
280 181 279 144 197			1	3	0.9
280 184 271 142 197			4		0.9
268 178 271 142 197			1	2	0.7
274 178 275 144 197				3	0.7
274 178 279 128 213		1	2		0.7
274 181 273 136 197	2		1		0.7
274 181 279 142 197			3		0.7
274 190 275 144 205				3	0.7
280 184 285 142 197			2	1	0.7
298 178 275 144 197				3	0.7
268 181 265 144 197			2		0.5
274 172 271 142 197				2	0.5
274 175 271 150 197	1	1			0.5
274 178 277 144 221			2		0.5
274 181 269 144 197			2		0.5
274 181 271 128 201	2				0.5
274 181 271 138 197			2		0.5
274 181 271 146 197		1	1		0.5
274 181 275 150 197			2		0.5
274 181 275 154 213				2	0.5
274 181 281 138 197			2		0.5
274 181 291 132 225			2		0.5
274 184 283 126 197			1	1	0.5
274 184 283 138 229			2		0.5
274 190 269 142 197			2		0.5
274 190 271 142 217				2	0.5
274 190 277 144 197			1	1	0.5
280 181 271 142 197		1		1	0.5
280 181 277 142 197				2	0.5
280 181 281 142 197			1	1	0.5

The first column shows the haplotypes defined by a contiguous string of PCR fragment sizes in base pairs for five neighbouring loci within this region. The following columns show the number of isolates from each year with the haplotype. The last column presents the frequencies for the entire population

The frequency of shared haplotypes was higher in later years; 8.9, 19.4, 60.8 and 51.3 % for 1984, 1991, 2008 and 2014 respectively. Using loci with peak LD as focus (SSR13 and SSR21), two long-range LD tracks of ~100 kb each could be distinguished (Additional file 5). The block determined by SSR13 was present in the earliest population sample from 1984 (Additional file 6). The types and the frequencies of these haplotypes increased in the more recent populations from 2008 and 2014. The haplotypes defined by SSR21 were dominant from 2008 and maintained a similar structure in 2014.

## Discussion

This is the first study describing extended haplotypes of microsatellite loci within a chromosome 6 region of *P. falciparum* previously determined with SNPs to be under selection. The microsatellite alleles derived from Illumina short read sequence data of clinical isolates collected in 2008 detected a 200 kb window of significantly elevated linkage disequilibrium that overlaps with the previously determined selective signature on the chromosome [6, 7, 16]. It shows microsatellites and linkage disequilibrium to be sensitive in detecting regions under selection as reported previously [14, 17]. The use of microsatellites as markers has the potential to focus on very recent events. In contrast to SNPs, Higher mutation rates will also provide sufficient variation in closely related populations, and would reveal only recent selective sweeps. As our focus here was over a relatively short period (23 years), any deviations in variability in the linked loci will be marking selection closer to the target locus. Several long-range haplotypes were detected in the sweep region suggesting more than a single founder haplotype under selection. To validate and examine haplotype structure and frequencies in this genomic region, four populations of *P. falciparum* isolates from The Gambia spanning 30 years (1984–2014) were characterized with a subset of 13 loci across 179.5 kb. During the 30 years, the first line chemotherapy for malaria changed from chloroquine to SP (2002) and then ACT (2008). These loci were highly diverse in all temporal *P. falciparum* populations genotyped. Similar to genomic loci from Illumina short reads, we detected strong linkage disequilibrium and several extended haplotypes. Sweeps lead to increase in LD when they are still in progress [18]. Thus, selection could have started as early as 1984 and continued into recent populations. This is also supported by increase in the frequencies of long-range haplotypes in the region from 1984 to recent populations. However, for the most common combination of multilocus alleles (from 1,135,849–1,207,757 bp), only two haplotypes found in the earliest population (1984) were present in single isolates from 1991 and 2008. On the other hand, other haplotypes upstream of this region were already at high frequencies in 1984. This would suggest selection on more than one locus or more than one event occurring prior to the 80s and between 80s and 90s. Antifolate anti-malarials were not officially used earlier than the year 2002 in the Gambia and so selection around these earlier years cannot be due to SP. The results here are against suggestions that anti-malarial SP might be the driver of selection in this region [5].

Finding a variety of long-range haplotypes at low to moderate frequencies in all temporal populations is contrary to the classical outcome of hard selective sweeps, where one haplotype carrying the favourable mutation rises in frequency to fixation [19]. With 36 genes coded for by the region with the selective signature, selection could be polygenic or both diversifying and directional selection may be acting on different loci in the region. While identifying the exact loci being selected remain beyond this current analysis, three genes within the region might be under non-neutral variation from previous SNP analysis and the analysis herein. The first gene is the acetyl-CoA synthetase (PF3D7_0627800) which was predicted as being under balancing selection [16]. Balancing selection increases diversity around a selected locus and would drive the multiplicity of haplotypes. Long-term maintenance of such polymorphisms and reduction in recombination would increase LD in neutral neighbouring loci around the focus of selection. The second gene in the region is 6-pyruvoyltetrahydropterin synthase, PTPS (PF3D7_0628000) which has a direct role in pterin metabolism and folate salvage pathways in *P. falciparum* [20]. Folate salvage is a major mechanism of SP resistance. The increase in complete long-range haplotype frequencies in the 2008 population provides support to selection from sulfonamides. However, haplotypes around the PTPS gene that is involved in sulfonamide metabolic processes seemed to have emerged earlier than 2002. Since SP was not in use when the earlier populations here were collected, selection of such pathways might be as a result of non-antimalarial sulfonamides, such as sulfamethoxazole/trimethoprim (cotrimoxazole), that have been used across Africa for a long time. Thirdly there is a phospholipase A2, PF3D7_0629300 (phosphatidylcholine-sterol acyltransferase, putative gene), which by Bayesian analysis of Fst distribution of ssr loci, may be under selection. The repeat locus within this gene showed the highest Fst and LD. Phospholipase activity increases membrane permeability and stimulation of many enzymes associated with processes that disrupt cytoskeleton and membrane structure [21]. They are also strongly inhibited by quinoline anti-malarials [22, 23]. Hence, the lipase may also be under directional selection from quinolines used in the 1990s. Chloroquine in particular was the most widely used quinolines against malaria before the 90 s and could have been driving selection. Antifolate SP replaced chlorquine from 2002 to 2008.

There was increase in frequencies of partial forms of haplotypes in the 2014 population, 6 and 12 years after the withdrawal of SP and chloroquine respectively as first line treatment. However, SP is still in use for intermittent preventive treatment and is now a component of seasonal malaria chemoprophylaxis [24]. The non-antimalarials antibiotics cotrimoxazole and derived quinolines were introduced in clinical practice in the 1960s while most quinoline antibiotics came into use in the 1980s. Cotrimoxazole in particular has been shown to be efficacious against malaria and thus might have been a source of selection on folate metabolism pathways for over half a century. If selection is acting on the phospholipase, due to quinolones, this could also explain the early origin of long-range haplotypes. Moreover, anti-malarial quinolines (e.g., quinine) have been in use against malaria for over is 400 years [25]. With SP being a component of SMC, increased frequencies of the sweep haplotypes in future analysis of SMC treated populations could confirm its role in selection. Meanwhile, analysis of populations with the earliest records of quinolone resistance such as Latin America could help resolve the involvement of quinolones as a selective force on this genomic region. With increasing accumulation of genomic data, this will be possible in the near future.

## Conclusions

This study has observed multiple long-range haplotypes of a region on *P. falciparum* chromosome 6 maintained over more than two decades in The Gambia. The identity of the target of selection remains unknown as the narrowest region implicated contains 24 genes involved in various metabolic processes. It is unlikely to be a result of anti-malarial drug selection only, as extended haplotypes existed before the widespread use of antifolates for malaria treatment, and before chloroquine resistance alleles had become locally common. It is possible that off-target drug action from antibiotics may have been responsible, or some completely different process may be maintaining extended haplotypes in this region of the parasite genome.

## Additional files

**Additional file 1.** Additional tables of raw illumina derived genotypes, repeat motif distribution, microsatellite fragment sizes and analysis, PCR primers and probes.

**Additional file 2.** Correlation of repeat length polymorphisms determined from Illumina short sequence reads and PCR fragment size analyses for three microsatellite loci (SSR1, SSR9 and SSR15) on chromosome 6 of *P. falciparum*. Each point is the Illumina repeat length (y-axis) plotted against the PCR fragment size (x-axis) in base pairs for a sample. The trend in correlation is shown as a broken blue line.

**Additional file 3.** Mean haplotype frequencies for microsatellite loci within windows of 10 kb across chromosome 6. The smoothed line is derived from haplotype frequency averages between adjacent windows plotted against the physical position on the chromosome. A total of 775 microsatellite loci were analysed. There were elevated haplotype frequencies spanning a 100 kb region that overlaps with the previously determined chromosome selective signature.

**Additional file 4.** Bayesian analysis of Fst distribution for 13 chromosome 6 microsatellites genotyped in 4 temporal populations in The Gambia. (a) Simulated WC –Fst is plotted against q-values following 10,000 iterations in Bayescan. Seven out of 13 loci (unshaded points) are under non-neutral variation at 5% false discovery rate. The locus with the highest positive deviation is at position 1207 kb in the phospholipase gene. (b) Distribution of Fst against a derivative of heterozygosity. Loci beyond the 95 % confidence interval for the distribution of Fst and heterozygosity are candidate targets of possible selection.

**Additional file 5.** Linkage disequilibrium map for 13 microsatellite loci across 179.5 kb of *P. falciparum* chromosome 6. Each plot presents data from a single population labelled at the top left of the plot panel with the year the isolates were collected. Loci are labelled at the top of each plot. Boxes in each plot represents LD between loci in blue or red indicating non-significant or highly significant r^2^ values between alleles of the loci. More intense colours represent extreme values of significance.

**Additional file 6.** Haplotype homozygosity across 179.5 kb region of chromosome 6 determined from 13 microsatellite loci by fragment analysis of temporal populations from the Gambia. Each plot shows bifurcation of haplotypes from the core locus with the thickness of the blue shaded lines corresponding to the frequency of the long-range haplotype. The horizontal axis shows the physical positions on the chromosome while the broken vertical line marks the position of the focal locus from which extended haplotypes were defined to the left and right. Plots on the left column were derived from haplotype structures at SSR13 (1,135,849 bp) for each population from 1984, 1991, 2008 and 2014 in rows from top to bottom and labelled on the right. The right column of plots present extended haplotypes derived from SSR21 (1,207,757 bp) for same populations. SSR13 and SSR21 showed the lowest and highest Fst values respectively within the region of elevated linkage disequilibrium.
