# Supplementary figures and images for: Exceptionally long-range haplotypes in Plasmodium falciparum chromosome 6 maintained in an endemic African population
Source: Malar J. 2016 Oct 21;15:515. doi: 10.1186/s12936-016-1560-7 (PMC5073846; doi:10.1186/s12936-016-1560-7)

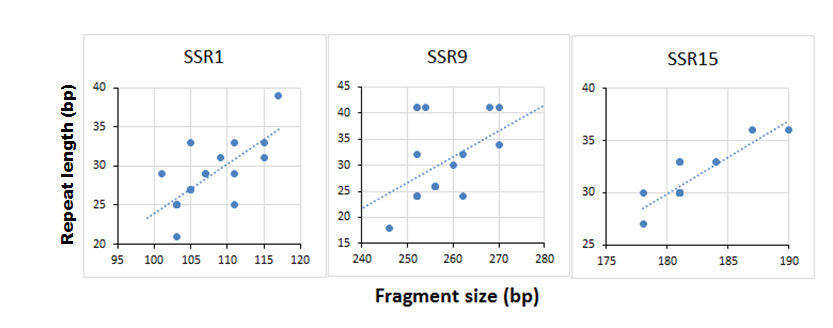

Supplement: Supplementary file 2 — Additional file 2. Correlation of repeat length polymorphisms determined from Illumina short sequence reads and PCR fragment size analyses for three microsatellite loci (SSR1, SSR9 and SSR15) on chromosome 6 of P. falciparum. Each point is the Illumina repeat length (y-axis) plotted against the PCR fragment size (x-axis) in base pairs for a sample. The trend in correlation is shown as a broken blue line. [file 12936_2016_1560_MOESM2_ESM.png]

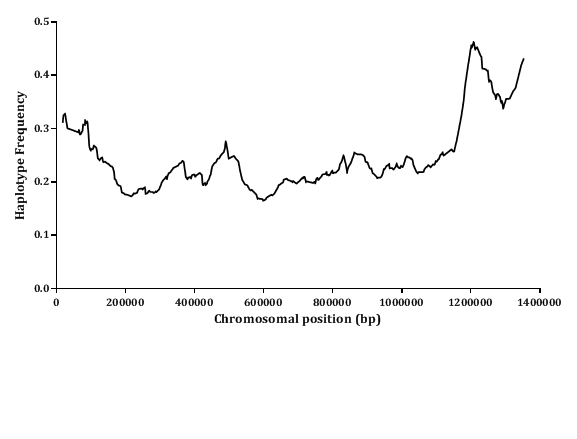

Supplement: Supplementary file 3 — Additional file 3. Mean haplotype frequencies for microsatellite loci within windows of 10 kb across chromosome 6. The smoothed line is derived from haplotype frequency averages between adjacent windows plotted against the physical position on the chromosome. A total of 775 microsatellite loci were analysed. There were elevated haplotype frequencies spanning a 100 kb region that overlaps with the previously determined chromosome selective signature. [file 12936_2016_1560_MOESM3_ESM.png]

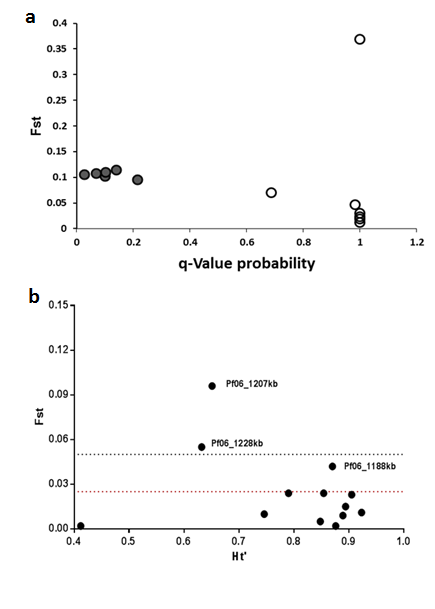

Supplement: Supplementary file 4 — Additional file 4. Bayesian analysis of Fst distribution for 13 chromosome 6 microsatellites genotyped in 4 temporal populations in The Gambia. (a) Simulated WC –Fst is plotted against q-values following 10,000 iterations in Bayescan. Seven out of 13 loci (unshaded points) are under non-neutral variation at 5% false discovery rate. The locus with the highest positive deviation is at position 1207 kb in the phospholipase gene. (b) Distribution of Fst against a derivative of heterozygosity. Loci beyond the 95 % confidence interval for the distribution of Fst and heterozygosity are candidate targets of possible selection. [file 12936_2016_1560_MOESM4_ESM.png]

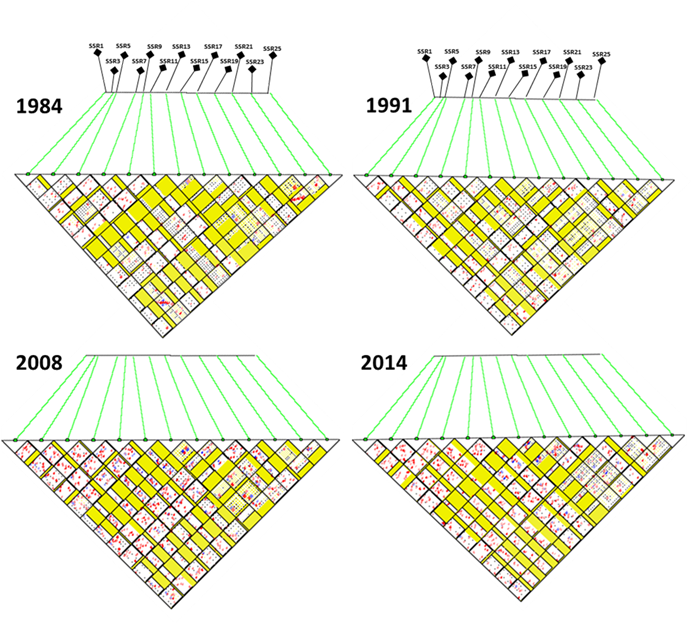

Supplement: Supplementary file 5 — Additional file 5. Linkage disequilibrium map for 13 microsatellite loci across 179.5 kb of P. falciparum chromosome 6. Each plot presents data from a single population labelled at the top left of the plot panel with the year the isolates were collected. Loci are labelled at the top of each plot. Boxes in each plot represents LD between loci in blue or red indicating non-significant or highly significant r2 values between alleles of the loci. More intense colours represent extreme values of significance. [file 12936_2016_1560_MOESM5_ESM.png]

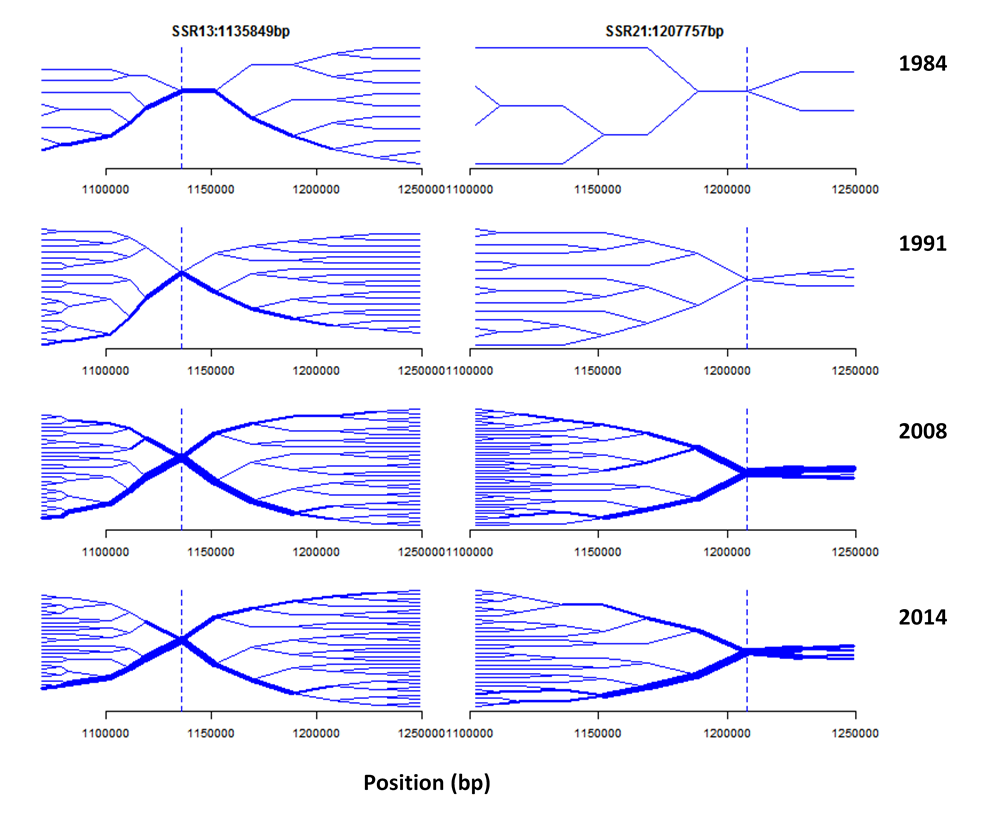

Supplement: Supplementary file 6 — Additional file 6. Haplotype homozygosity across 179.5 kb region of chromosome 6 determined from 13 microsatellite loci by fragment analysis of temporal populations from the Gambia. Each plot shows bifurcation of haplotypes from the core locus with the thickness of the blue shaded lines corresponding to the frequency of the long-range haplotype. The horizontal axis shows the physical positions on the chromosome while the broken vertical line marks the position of the focal locus from which extended haplotypes were defined to the left and right. Plots on the left column were derived from haplotype structures at SSR13 (1,135,849 bp) for each population from 1984, 1991, 2008 and 2014 in rows from top to bottom and labelled on the right. The right column of plots present extended haplotypes derived from SSR21 (1,207,757 bp) for same populations. SSR13 and SSR21 showed the lowest and highest Fst values respectively within the region of elevated linkage disequilibrium. [file 12936_2016_1560_MOESM6_ESM.png]
